# Preparation of Silica Aerogel/Resin Composites and Their Application in Dental Restorative Materials

**DOI:** 10.3390/molecules27144414

**Published:** 2022-07-09

**Authors:** Jingru Cheng, Yuyi Deng, Yujin Tan, Jiawei Li, Yongsheng Fei, Congcong Wang, Jingjing Zhang, Chenxi Niu, Qian Fu, Lingbin Lu

**Affiliations:** Special Glass Key Lab of Hainan Province, School of Materials Science and Engineering, Hainan University, Haikou 570228, China; cheng_jingru617@163.com (J.C.); dengyuyiyi@163.com (Y.D.); tyj1060562383@163.com (Y.T.); yx163lijiawei@163.com (J.L.); fei_yongsheng21@163.com (Y.F.); wangcongkf@163.com (C.W.); jingjing_cheung@126.com (J.Z.); ncx13027683758@163.com (C.N.); fuqian1668@163.com (Q.F.)

**Keywords:** silica aerogel, dental restorative materials, mechanical properties, antibacterial capabilities

## Abstract

As the most advanced aerogel material, silica aerogel has had transformative industrial impacts. However, the use of silica aerogel is currently limited to the field of thermal insulation materials, so it is urgent to expand its application into other fields. In this work, silica aerogel/resin composites were successfully prepared by combining silica aerogel with a resin matrix for dental restoration. The applications of this material in the field of dental restoration, as well as its performance, are discussed in depth. It was demonstrated that, when the ratio of the resin matrix Bis-GMA to TEGDMA was 1:1, and the content of silica aerogel with 50 μm particle size was 12.5%, the composite achieved excellent mechanical properties. The flexural strength of the silica aerogel/resin composite reached 62.9546 MPa, which was more than five times that of the pure resin. Due to the presence of the silica aerogel, the composite also demonstrated outstanding antibacterial capabilities, meeting the demand for antimicrobial properties in dental materials. This work successfully investigated the prospect of using commercially available silica aerogels in dental restorative materials; we provide an easy method for using silica aerogels as dental restorative materials, as well as a reference for their application in the field of biomedical materials.

## 1. Introduction

Dental caries is one of the most widespread diseases among human beings, and improper treatment can lead to many adverse consequences [1]. Potential mechanisms of this disease include demineralization caused by acid erosion of bacteria in the dental biofilm, diets rich in fermentable carbohydrates, and poor oral hygiene, which may contribute to the growth of such acid-producing bacteria in the biofilm [2,3]. As a tissue with a weak material metabolism and energy metabolism, once damaged, hard dental tissue cannot regenerate and repair itself [4]. To restore the normal shape and function of the teeth, restorative dentistry is the only effective solution.

To obtain high-performing dental restorative materials, researchers have conducted extensive investigations. The filling material used in early dental restoration materials is a silver amalgam material, which has excellent mechanical properties and a long life span, and can therefore meet people’s basic chewing needs. However, it was gradually phased out due to the fact that its appearance did not match that of the natural teeth, and because of mercury toxicity and other disadvantages [5,6,7]. Composite resin-based filling materials are mainly composed of an organic resin matrix, diluent, inorganic filler, and a photoinitiator, which are the most popular dental filling materials at present [8,9,10]. The composite resin triggers in situ polymerization by blue light irradiation, and this reaction can fill in the dental cavity directly, in order to match the defective shape well and restore the missing dentin/enamel structure [11]. Moreover, the composite resin is popular because of its similarity in color to dentin, and because it is relatively easy to handle.

Among the numerous dental resin monomers, the resin monomer system consisting of bisphenol A-glycidyl methacrylate (Bis-GMA) and triethylene glycol dimethacrylate (TEGDMA) is the most commonly used type of synthetic composite resin [12,13,14]. Methacrylate monomers contain alkenyl with a carbon–carbon double bond (C=C) functional group, which contributes to the rapid chain polymerization and cross-linking of polymers. Nevertheless, pure resin materials are not mechanically strong enough on their own, and often require inorganic filler compounds to enhance their mechanical and physical properties. Inorganic filler materials can enable significant improvements in the strength, modulus [15,16], surface hardness [17,18], aesthetics [19], polymerization shrinkage and stress [20], and antimicrobial activity of resin materials [21,22]. The commonly used inorganic fillers with particle sizes ranging from 5 nm to 500 μm are mainly silica-based particles [21,23,24,25,26,27], glass ceramics [28,29], metals [9,30,31], and mineral particles [32,33,34,35]. However, these commonly used composite resin materials still suffer from poor mechanical properties, poor antimicrobial properties, and restricted clinical service life. Furthermore, the composite resin materials currently used for dental restorations have been reported to have annual failure rates as high as 3–11% [36,37,38]. Additionally, inorganic fillers often need multiple components, which leads to high costs, poor operational convenience, and difficulties in performance control. Therefore, much attention has been paid to the discovery and application of new fillers. For instance, Yadav et al. investigated the effect of inorganic fillers of nano-hydroxyapatite with micron alumina and titanium oxide on the physical, chemical, and mechanical properties of two dental resin composites (DHA and DHT) and found that the restorative effect of the DHA composite was superior to that of DHT dental composite [30]. Yang et al. enhanced the structure of spray-dried silica colloidal nanoparticle clusters (SCNC) by roasting and combining them with different fillers to achieve an excellent overall performance of dental restorative materials [26].

Silica aerogel is a new kind of silicon-based nanomaterial, which has the advantages of high specific surface area and high porosity. It is also the most popular aerogel material that has been industrially produced, but its application is currently only concentrated in the field of thermal insulation materials, which limits its development. Thus, the extension of its application is urgently required. In fact, although the pure silica aerogel block is fragile, the powdered silica aerogel is a favorable mechanical reinforcement material.

With the goal of broadening the practical applications of silica aerogel, this study took as its research object the commercially available silica aerogel, which was compounded with a dental resin matrix; this enabled us to study the influence of the introduction of commercially available silica aerogel powder on the performance of the dental resin matrix, and to explore the feasibility of its application in the field of dental restoration materials.

## 2. Results

### 2.1. Microstructure Analysis of Silica Aerogel/Resin Composites

Inorganic fillers with a high specific surface area, high porosity, a mesoporous structure, and other desirable properties have been proven to improve the mechanical properties of dental restoration materials [39]. Silica aerogel has the characteristics of high specific surface area, high porosity, and a mesoporous structure, making it a promising potential dental restorative filler. The silica aerogels used in the reported studies were usually customized according to the research requirements. These methods and properties are not the same as those of commercially available silica aerogels. In order to draw conclusions that have direct relevance to market applications, three commercially available silica aerogel commodities were used in this study, in order to investigate their feasibility and methods as dental restorative fillers.

**Figure 1:**
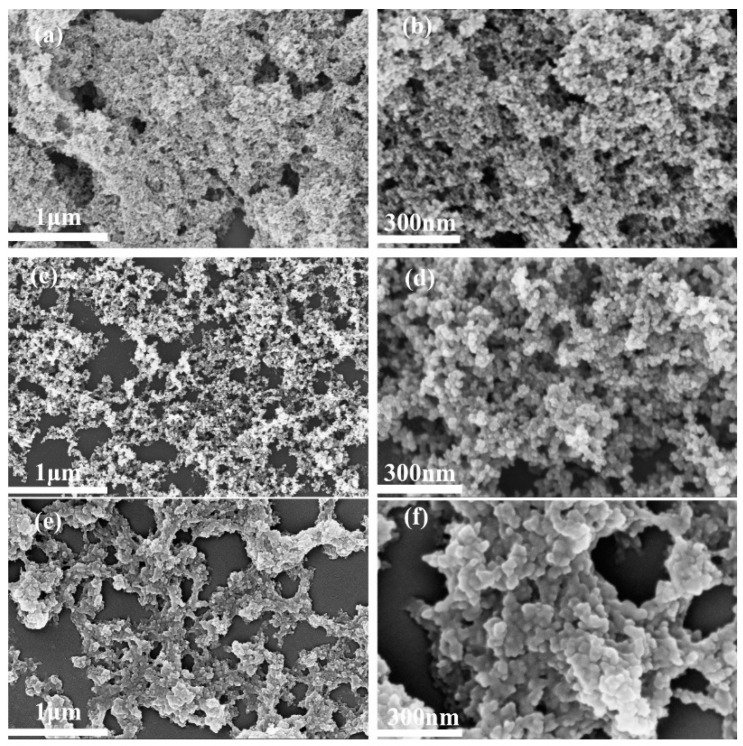
FESEM diagrams of silica aerogels with different particle sizes: 15 μm (**a**,**b**), 30 μm (**c**,**d**), 50 μm (**e**,**f**).

**Figure 2:**
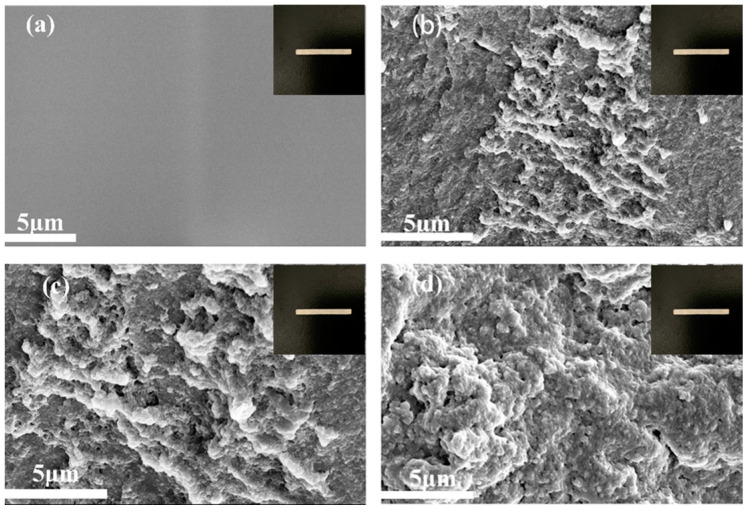
FESEM images of silica aerogel/resin composites before and after adding silica aerogel filler: (**a**) SA/R-0, (**b**) SA/R-5, (**c**) SA/R-6, (**d**) SA/R-2.

Shown in Figure 1a–f and Figure S1 are the FESEM images of the three commercially available silica aerogel powders, which have particle sizes of 15 μm, 30 μm, and 50 μm, respectively. The findings showed that the three silica aerogels appear to have a relatively regular morphology with good dispersion and abundant pore structure. The silica aerogel/resin composites were obtained by directly blending and shaping with the silica aerogels and the resin matrix individually. As seen in Figure 2, all three silica aerogel powders were uniformly dispersed in the resin matrix without agglomeration. The rich pores possessed by the silica aerogels offered an interpenetrating filler–matrix system for the resin matrix. The resin matrix could fully penetrate in the silica pores and complete cross-linking and curing, resulting in a ‘micro-mechanical interlocking’ effect at the interface of the resin and aerogel particles. This effect had a significant impact on improving the mechanical strength of the composite [40].

### 2.2. Analysis of the Chemical Structure of the Silica Aerogel/Resin Composites

**Figure 3:**
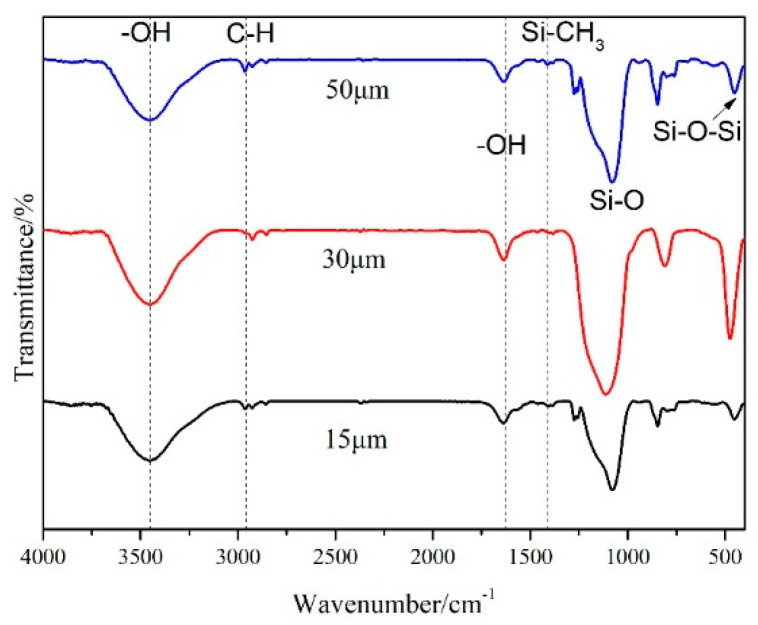
Infrared spectra of silica aerogels with different particle sizes.

Figure 3 shows the infrared spectroscopy of three commercially available silica aerogel products. As shown in Figure 3, the infrared spectroscopies are basically the same across the three products. The characteristic peaks that appear at 3450 and 1630 cm^−1^ are the vibrational stretching peaks of -OH, caused by the water molecules absorbed from the environment [41]. The distinctive peak at 1078 cm^−1^ is the symmetric stretching vibration peak of the Si-O bond. The absorption peaks corresponding to the characteristic bands of asymmetric stretching vibration of C-H and asymmetric bending vibrations in Si-CH_3_ are assigned at 2961 and 1411 cm^−1^, respectively. The absorption peaks near to 450 cm^−1^ are caused by the bending vibration of Si-O-Si [42]. It can be seen that, before leaving the factory, the three products were hydrophobic with a stable silicyl group, so that they could be stably preserved to prevent moisture absorption and agglomeration. Further, the silica aerogel with both the hydrophilic groups -OH and the hydrophobic -CH_3_ was apt to blend evenly with the resin. At the same time, the wettability of the resin was not obviously affected by the aerogel, since this resin accounts for the main proportion of the material.

**Figure 4:**
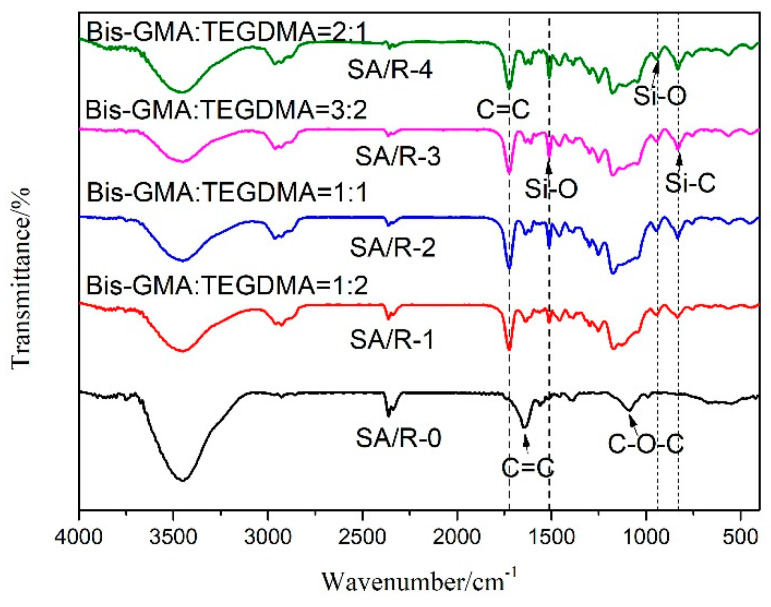
Infrared spectra of the silica aerogel/resin composites with different Bis-GMA: TEGDMA.

**Scheme 1:**
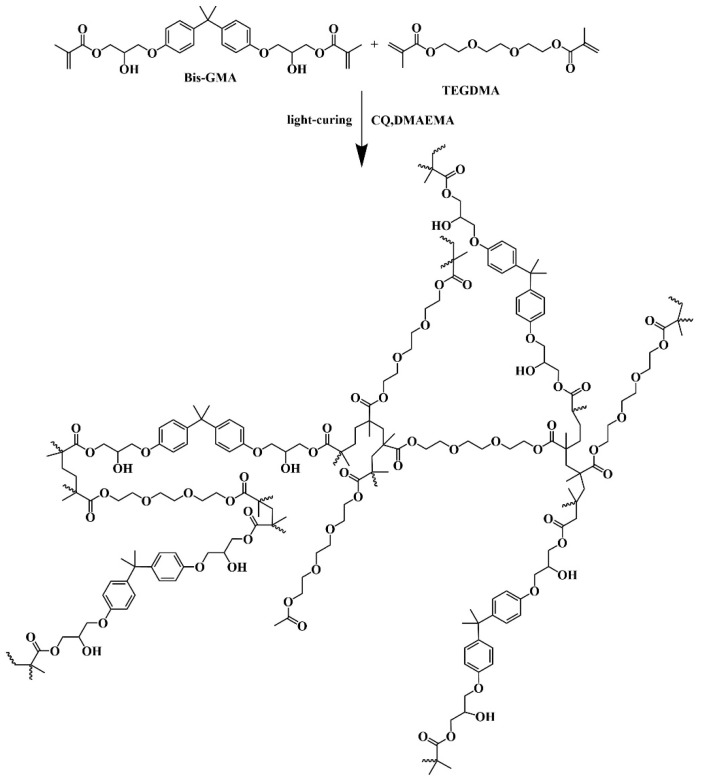
The mechanism of radical copolymerization between Bis-GMA and TEGDMA.

Figure 4 depicts the infrared spectroscopy of the silica aerogel/resin composites with various resin monomer ratios. The infrared spectroscopy of the composites shows obvious differences compared to the SA/R-0 without silica. From SA/R-1 to SA/R-4, the ratio of Bis-GMA to TEGDMA rose in steps of 1:2, 1:1, 3:2, and 2:1. There were many C=C bonds and benzene ring structures, as shown in Scheme 1. When the silica aerogel was blended with the resin matrix, the stretching vibration peak of the C=C bond in the resin shifted significantly: from 1640 cm^−1^ to 1723 cm^−1^, a red shift occurred. This indicated that the silica aerogel and the resin matrix were not simply physically blended, but possibly formed a hydrogen bond interaction. This led to a stronger interaction between them, which facilitated the enhancement of the composite’s mechanical properties. Considering that this peak of the composites was formed after the addition of silica, 1512 cm^−1^ might be the benzene ring skeleton vibration peak caused by the Si-O group on the surface of silica. The peak became stronger as the amount of Bis-GMA increased. After the Bis-GMA content increased to 1:1, there was no significant change in the intensity of the peak. This was due to the fact that the amount of silica was fixed in these composites, and the interaction between resin and silica aerogel was at its limit. The data suggest that the structure of the resin matrix tended to be stable, and the interaction between the resin and silica aerogel was at its strongest when the ratio of Bis-GMA to TEGDMA was 1:1. The stretching vibration peak of C-O-C of the ester group in SA/R-0, 1085 cm^−1^, was covered by the symmetric stretching vibration peak of the Si-O group in the silica. At the same time, new characteristic peaks for the composites were apparent at 940 cm^−1^ and 830 cm^−1^, which were the stretching vibration peaks of Si-O and Si-C.

### 2.3. Analysis of the Thermal Stability Properties of Silica Aerogel/Resin Composites

**Figure 5:**
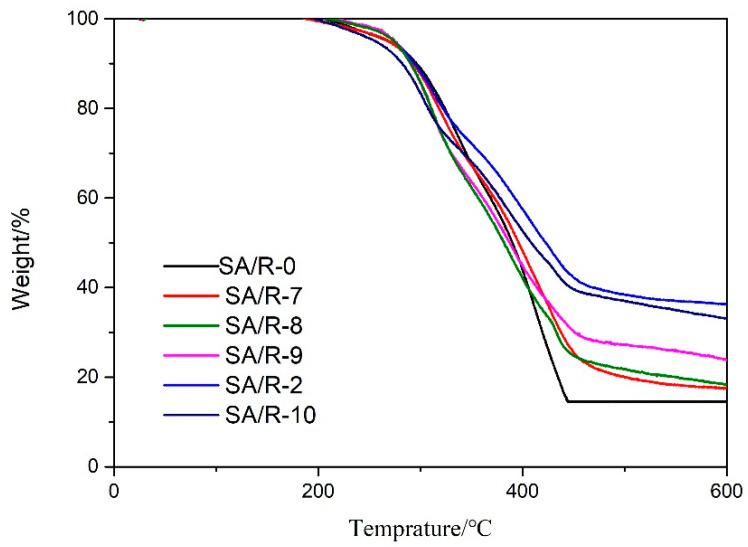
TG curves of silica aerogel/resin composites with different contents of silica aerogel.

Figure 5 shows the thermogravimetric curves for the composites with different silica aerosol contents. The temperature of the pure resin was 268 °C at 5% weight loss, and the mass stabilized at 445 °C when the pyrolysis process was complete. At 5% weight loss, the temperatures of silica aerogel/resin composites with silica aerogel contents of 5%, 7.5%, 10%, 12.5%, and 15% were at 269 °C, 274 °C, 274 °C, 275 °C, and 256 °C, respectively, corresponding to mass residuals at the termination of thermal decomposition of 17.596%, 18.406%, 23.933%, 36.324%, and 33.179%. In comparison, the final mass residual of the pure resin material was only 14.526%. This shows that the thermal stability of the composites with silica aerogel is superior to that of the pure resin material. Increasing the content of silica aerogel was beneficial to improving the thermal stability of the composite resin to a certain extent. The best thermal stability of the silica aerogel/resin composite was achieved at a content of 12.5%. Considering the above results, we can speculate that the improvement in the thermal stability of the composites is due to the ‘micro-mechanical interlocking’ effect between the resin and aerogel particles and the hydrogen bonding between the groups.

### 2.4. Effect of Silica Aerogel on the Mechanical Properties of Silica Aerogel/Resin Composites

The short life span of dental restorative materials is mainly due to stress fractures. Consequently, it is imperative to increase the fracture stress of these materials in order to extend their lifespans.

**Figure 6:**
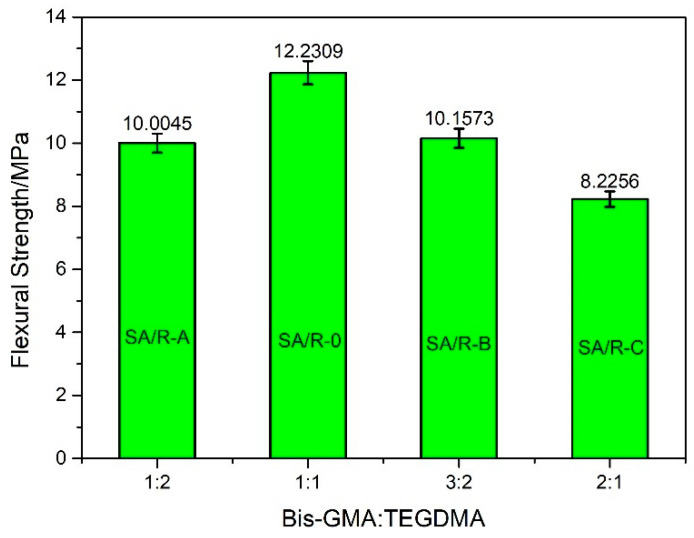
The flexural strength of blank samples, without silica aerogel, with different Bis-GMA: TEGDMA.

**Figure 7:**
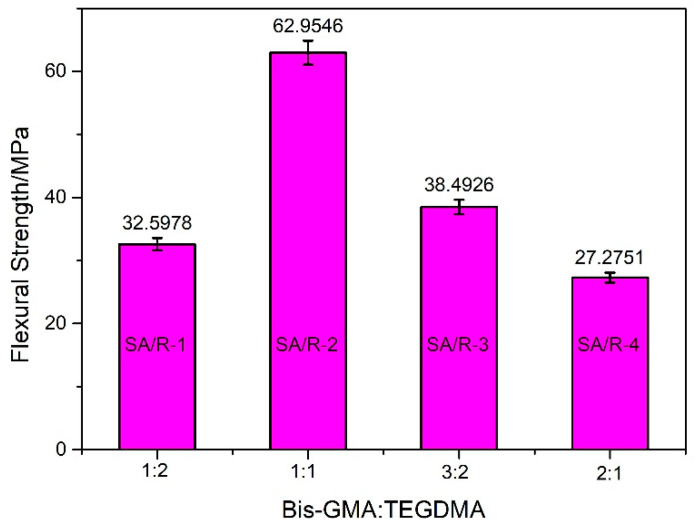
The flexural strength of silica aerogel/resin composites with different Bis-GMA: TEGDMA.

The ratio of two organic monomers in the resin matrix affects the mechanical properties of the final cured products, to some extent. A silica aerogel content of 50 μm was chosen to investigate the effect of the ratio of Bis-GMA to TEGDMA in the resin matrix on the flexural strength of the silica aerogel/resin composite with a fixed aerogel filler content of 12.5%. With the increase in the Bis-GMA:TEGDMA ratio of the pure resin, the flexural strength showed a trend of first increasing and then decreasing, as is clearly shown in Figure 6. This trend was similar to that observed in the composites, as shown in Figure 7. As can be observed from this Figure 7, the maximum flexural strength was 62.9546 MPa when the Bis-GMA:TEGDMA ratio was 1:1. It benefited from the strongest interaction between Bis-GMA and TEGDMA, as shown in Figure 4. This ratio was adopted in the subsequent experiments to investigate the effect law of silica aerogel powder on the mechanical properties of the composites.

**Figure 8:**
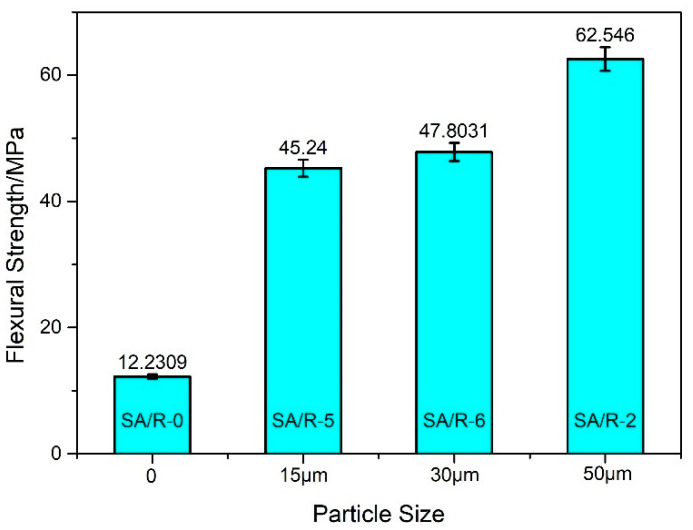
Effect of the particle size of silica aerogel powder on the flexural strength of the silica aerogel/resin composites.

**Figure 9:**
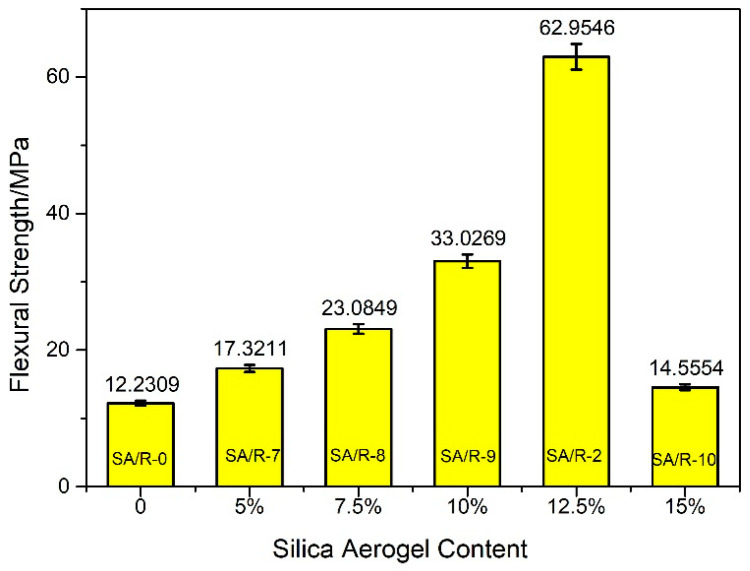
Effect of silica aerogel content on the flexural strength of the silica aerogel/resin composites.

As shown in Figure 8, the flexural strength of the pure resin was only 12.2309 MPa, but the flexural strength was significantly increased with the addition of silica aerogel. The flexural strength of the silica aerogel/resin composite with 50 μm of silica aerogel was more than five times that of the pure resin. Hence, silica aerogel with a particle size of 50 μm is the most effective means of enhancing the flexural properties of the composites. Within a certain range, flexural strength was improved as the filler content increased. The flexural strength of the silica aerogel/resin composite was at its highest with 12.5% filler content. This can be attributed to the fact that, on the one hand, silica aerogel has a unique penetrating pore structure, which can form a ‘micro-mechanical interlocking’ effect by the interpenetration of matrix and filler through resin monomer diffusion, thus improving the interfacial bonding state, and leading to the improved macroscopic mechanical properties of the composites [39]. On the other hand, the rich pores and large specific surface area of silica aerogel increase the contact area between resin matrix and nanoparticles, which further enhances the strength of the composites [43]. Simultaneously, the hydrogen bonds between the resin matrix and the silica aerogel further improved the bonding strength between them and enhanced their mechanical properties. In addition, the introduction of excessive powder materials can cause the phase separation phenomenon between the organic and inorganic phases, which might destroy the material’s overall structural homogeneity and performance stability, thereby leading to a decrease in flexural strength. As Figure 9 shows, the flexural strength of the silica aerogel/resin composite with 15% filler content was significantly decreased. Therefore, 12.5% silica aerogel with a particle size of 50 μm is the most effective at enhancing the flexural properties of the composites.

### 2.5. Analysis of the Water Sorption and Solubility of the Silica Aerogel/Resin Composites

In order to evaluate the stability of the materials, their water absorption and solubility were characterized. Water was used as a solvent in the solubility experiments. The results of the water absorption of the silica aerogel/resin composites are shown in Table 1.

As shown in Table 1, the water absorption of all of the composite materials was acceptable. Compared to the pure resin sample SA/R-0, the water sorption of silica aerogel/resin composites was obviously decreased. With the increase in silica aerogel content, water absorption was gradually reduced. This was mainly attributed to the increased hydrophobic groups of silica aerogel. At the same time, due to the penetration between the resin matrix and the silica aerogel, there were few pore-like structures for water absorption.

**Figure 10:**
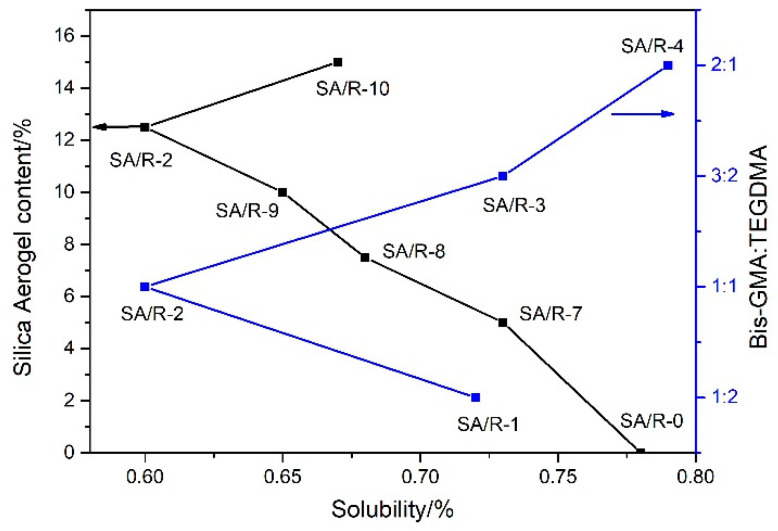
Solubility (SL) of silica aerogel/resin composites.

Solubility is mainly determined by the number of unreacted monomers [44]. As shown in Figure 10, the pure resin sample SA/R-0 had high solubility, which was due to the unreacted monomers released from the resin matrix. After water absorption, the resin swelled and the internal network structure became expanded, which was conducive to the dissolution of unreacted monomers. After being compounded with silica aerogel, the solubility of all of the composites decreased. It can be inferred that silica aerogel inhibited resin swelling by filling in the structure and interacting with the resin. The sample SA/R-2 with the optimum monomer ratio had the lowest solubility, which meant fewer unreacted monomers. The dissolution of silica aerogel is the other factor related to solubility. The sample SA/R-10 with the maximum silica aerogel showed increased solubility.

The resin expands after water absorption, which can make up for the polymerization shrinkage of the resin at a certain extent. However, high water absorption will reduce the mechanical properties of the material, resulting in the shortening of the service life. Exudative monomers may cause inflammation of pulp cells and surrounding tissues. [45] Therefore, dental restorative materials should not have high rates of water absorption and dissolution. Consequently, the comprehensive properties of the composite SA/R-2 are the best.

### 2.6. Analysis of Shore Hardness of Silica Aerogel/Resin Composites

The shore hardness test is the simplest and most common method for conducting hardness tests. Table 2 shows the hardness values of the samples, obtained by the shore test.

It can be seen from Table 2 that the hardness of the composites with silica aerogel was greater than that of the pure resin sample. It was indicated that the ‘micro-mechanical interlocking’ effect at the interface of resin and aerogel particles enhances their hardness, as well as their flexural strength. With the increase in silica aerogel, the hardness increased at first and then decreased. This is consistent with the trend observed in flexural strength.

### 2.7. Analysis of Antibacterial Properties of Silica Aerogel/Resin Composites

**Figure 11:**
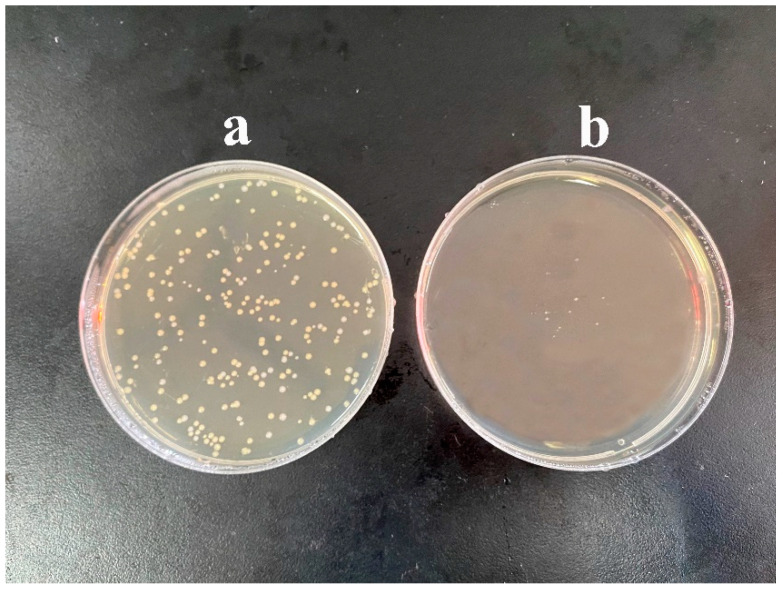
Agar medium inoculated with *Staphylococcus aureus* for 24 h ((**a**) SA/R-0 bacterial suspension, (**b**) SA/R-2 bacterial suspension)).

Acid attacks by bacteria in the oral cavity tend to shorten the service lives of dental restorative materials in the environments where they are used. To obtain a restorative material with an antimicrobial effect, extra antimicrobial components are frequently added, increasing the cost of the material and complicating the implementation process. In this work, it was found that the presence of silica aerogel unexpectedly exerted an antibacterial function. Figure 11 depicts an agar medium dripped with an SA/R-0 bacterial suspension and an agar medium dripped with an SA/R-2 bacterial suspension. After 24 h, there was noticeable bacterial multiplication in sample a, but no visible bacterial multiplication was found in sample b. As a result, it can be concluded that the silica aerogel/resin composite has a clear antibacterial effect. It is assumed that the hydrophobic groups on the surface of the silica aerogel affected the permeability of microbial cell membranes, which impeded normal metabolic action and prevented bacteria growth, resulting in effective antimicrobial properties [46,47]. This finding indicates that, if silica aerogel were used in dental restorative materials, it would not only improve the mechanical properties of the resin materials, but also provide an antibacterial function at the same time. This would have a significant effect on extending the materials’ service lives and preventing the occurrence of secondary dental caries.

## 3. Conclusions

In this work, we used a commercially available silica aerogel as a filler material for dental restorations; using a simple method, we compounded it with a resin matrix to develop silica aerogel/resin composites with excellent mechanical properties and antibacterial functions. The effects of using silica aerogel as a filler in conjunction with the resin matrix were thoroughly investigated. This investigation demonstrated that silica aerogel can be uniformly mixed with resin matrix. The interpenetration and the mechanical interlocking between matrix and filler improve the interfacial bonding state, while, at the same time, the hydrogen bonding between the silica aerogel and the resin matrix strengthened the interaction between them. This effectively improved the macroscopic mechanical properties and thermal stability of the materials. The silica aerogel/resin composite acquired the best flexural strength when the ratio of resin monomer Bis-GMA to TEGDMA was 1:1, and 50 μm silica aerogel with 12.5% content was selected. Compared to pure resin, the silica aerogel/resin composite had lower water absorption and dissolution, higher hardness levels, and, at the same time, a considerable antibacterial effect. As a result, by introducing a single filler, this study both accomplished the mechanical improvement of the resin matrix, and achieved improved antibacterial activity. This research demonstrated the effectiveness of using commercial silica aerogel in the field of dental restorative materials, not only broadening the application possibilities of silica aerogel products in the biomedical field, but also developing a potential new dental restorative material.

## 4. Materials and Methods

### 4.1. Materials

Silica aerogel (15 μm, 50 μm) was purchased from Shenzhen Zhongning Technology Co., Ltd. (Shenzhen, Guangdong, China) Silica aerogel (30 μm) was purchased from Yichang Huifu Silica Material Co., Ltd. (Yichang, Hubei, China). All aerogels were hydrophobic modified. Anhydrous ethanol (AR, 99.7%) was purchased from Xilong Science Co., Ltd. (Guangzhou, Guangdong, China). Bisphenol A-glycidyl methacrylate (Bis-GMA) (AR) was purchased from Sigma-Aldrich (City of Saint Louis, MO, USA). Triethylene glycol dimethacrylate (TEGDMA) (95%), 2-(Dimethylamino) ethyl methacrylate (DMAEMA) (95%), and camphorquinone (CQ) (98%) were purchased from Maclean’s (Shanghai, China). Nutrient agar (BR) was purchased from Guangdong Huan Kai Microbial Technology Co., Ltd. (Guangzhou, Guangdong, China). *Staphylococcus aureus* (ATCC 6538) was purchased from the Beijing Biological Conservation Center (Beijing, China). Deionized water and sterile water were prepared in the laboratory.

### 4.2. Preparation of Silica Aerogel/Resin Composites

A resin system (BisGMA/TEGDMA, 49.5 wt/49.5 wt) with photo-initiators (CQ/DMAEMA, 0.5 wt/0.5 wt) was used as resin matrix for the preparation of the silica aerogel/resin composites. Specified amounts of Bis-GMA, TEGDMA, CQ, and DMAEMA were mixed well and sonicated for 3 min. The light curing lamp emits a blue light with an intensity of 1200~2000 mw/cm^2^. The light curing time was 120 s. Then, a given amount of silica aerogel powder was gradually blended into the resin matrix and mixed thoroughly to obtain a homogeneous paste. The paste was injected into the mold, irradiated with curing-light, and cured in a blue light environment to obtain the silica aerogel/resin composite (SA/R). The specific sample preparation parameters are shown in Table 3.

### 4.3. Bacteriostasis Experiment

#### 4.3.1. Preparation of the Culture Medium

First, 1000 mL of deionized water was added to a beaker and heated to 100 °C with a set electric thermostat. Then, 33 g of nutrient agar was weighed and dispersed evenly in the deionized water, and stirred until the agar powder was completely dissolved to obtain a clear yellow solution. The agar solution was divided into two 500 mL blue-capped bottles and placed into a portable autoclave steam sterilizer, with the parameters set at 121 °C and 1.5 MPa pressure, for 20 min. After the temperature of the autoclave dropped to about 60 °C, the agar solution was removed and placed on a clean bench for operation. Before the temperature of the agar solution dropped to room temperature, the agar solution was spread evenly in the petri dishes and left to stand until it solidified.

#### 4.3.2. Preparation of Bacterial Suspension

To obtain bacterial suspensions for use, an appropriate amount of a strain of *Staphylococcus aureus* (*S. aureus*) was gently scraped using a disposable inoculation loop, placed in sterile water, and diluted to 10^−7^ times.

#### 4.3.3. Antibacterial Test

*S. aureus* was selected as the strain for the analysis. The prepared silica aerogel/resin composite SA/R-2 was immersed in the bacterial suspension for 10 min, and shaken with an adjustable vortex mixer for 30 s. Then, 100 μL of the sample-soaked bacterial suspension was pipetted onto the solidified agar and applied evenly with the applicator stick until all the liquid penetrated into the agar. The agar medium was then placed in an incubator at 37 °C and incubated for 24 h, in order to observe the distribution of colonies on the surface of the medium. Meanwhile, the experiment was also conducted with SA/R-0 as the control group.

### 4.4. Characterization

A field emission scanning electron microscope (thermoscientific, Verios G4 UC, Waltham, MA, USA) was used to observe and analyze the microscopic morphology of the silica aerogel raw materials, the pure resin material samples, and the silica aerogel/resin composites. The samples were pre-treated with spraying gold and an accelerating voltage of 5 kV.

A fourier transform infrared spectrometer (BRUKER, T27, Bremen, Germany) was used to test the infrared spectroscopy of the samples. The samples were ground separately and mixed with potassium bromide. The spectrum range was set at 400–4000 cm^−1^.

The prepared samples were dried and ground into powders for thermal stability testing. The experiment was conducted using a comprehensive thermal analyzer (Jing Yi Gao Ke, ZCT-1, Beijing, China) in a nitrogen atmosphere. The heating rate was set to 10 ℃/min and the temperature range was set from room temperature to 600 ℃. The composites with 50 μm silica aerogel used as a filler were tested to characterize the effect of silica aerogel content on the thermal stability of the composite.

The silica aerogel/resin composites were prepared in the size of 25 mm × 2 mm × 2 mm, and the flexural strength of the samples was tested using a vertical electronic universal testing machine (Shimadzu, AGS-X, Kyoto, Japan). The crosshead loading speed was set to 1 mm/min, and 8 parallel samples were tested in each group. The flexural strength (*FS* (MPa)) of the samples was calculated based on the test results and Equation (1):

(1) $$\;\;\;\;\;\;FS=\frac{3PL}{2WT^{2}}$$

where *P* is the maximum flexural load (N), *L* is the span (mm) of the test, *W* is the width (mm) of the sample, and *T* is the thickness (mm) of the sample.

The determination of the water absorption and solubility of the samples was carried out using the following method. First, the initial mass *M*_1_ of each sample was measured by an analytical balance. Then, the samples were immersed in 30 mL deionized water at 37 ℃. At regular intervals, the samples were taken out and their surfaces wiped dry to weigh the mass of the samples. Then, the samples were immersed in deionized water again. The above operation was repeated until the mass of the samples did not change significantly, and the balance mass *M*_2_ was recorded. Finally, the samples were placed in a vacuum oven at 60 ℃ for drying, and weighed once a day until the mass of the samples remained stable: this mass was denoted as *M*_3_. Water absorption (*WS*) and solubility (*SL*) were calculated according to Formulas (2) and (3), respectively.

(2) $$WS=\frac{M_{2}-M_{3}}{M_{3}}\times 100\%$$

(3) $$SL=\frac{M_{1}-M_{3}}{M_{1}}\times 100\%$$

Shore hardness was measured using a shore hardness tester (Shanghai Yi Zong Precision Instrument Co., Ltd., LX-A, Shanghai, China).

## Figures and Tables

**Table 1:** Water sorption (WS) of silica aerogel/resin composites.

Sample	Silica Aerogel Content (%)	WS (%)
SA/R-0	0	1.85 ± 0.21
SA/R-7	5	0.48 ± 0.05
SA/R-8	7.5	0.44 ± 0.06
SA/R-9	10	0.37 ± 0.05
SA/R-2	12.5	0.30 ± 0.06
SA/R-10	15	0.26 ± 0.04

**Table 2:** Shore hardness of silica aerogel/resin composites.

Sample	Silica Aerogel Content (%)	Shore Hardness (A)
SA/R-0	0	70
SA/R-7	5	85
SA/R-8	7.5	95
SA/R-9	10	87
SA/R-2	12.5	100
SA/R-10	15	88

**Table 3:** Experimental parameters of silica aerogel/resin composites.

Sample	Bis-GMA:TEGDMA	Particle Sizes (μm)	SA (wt%)
SA/R-0	1:1	-	0
SA/R-1	1:2	50	12.5
SA/R-2	1:1	50	12.5
SA/R-3	3:2	50	12.5
SA/R-4	2:1	50	12.5
SA/R-5	1:1	15	12.5
SA/R-6	1:1	30	12.5
SA/R-7	1:1	50	5
SA/R-8	1:1	50	7.5
SA/R-9	1:1	50	10
SA/R-10	1:1	50	15
SA/R-A	1:2	-	0
SA/R-B	3:2	-	0
SA/R-C	2:1	-	0

## Data Availability

The data presented in this study are available on request from the corresponding author.

## Supplementary Materials

The following supporting information can be downloaded at: https://www.mdpi.com/article/10.3390/molecules27144414/s1, Figure S1: FESEM of silica aerogel/resin composites before adding silica aerogel filler.
