# Supplementary figures and images for: Preparation of Silica Aerogel/Resin Composites and Their Application in Dental Restorative Materials
Source: Molecules. 2022 Jul 9;27(14):4414. doi: 10.3390/molecules27144414 (PMC9323775; doi:10.3390/molecules27144414)

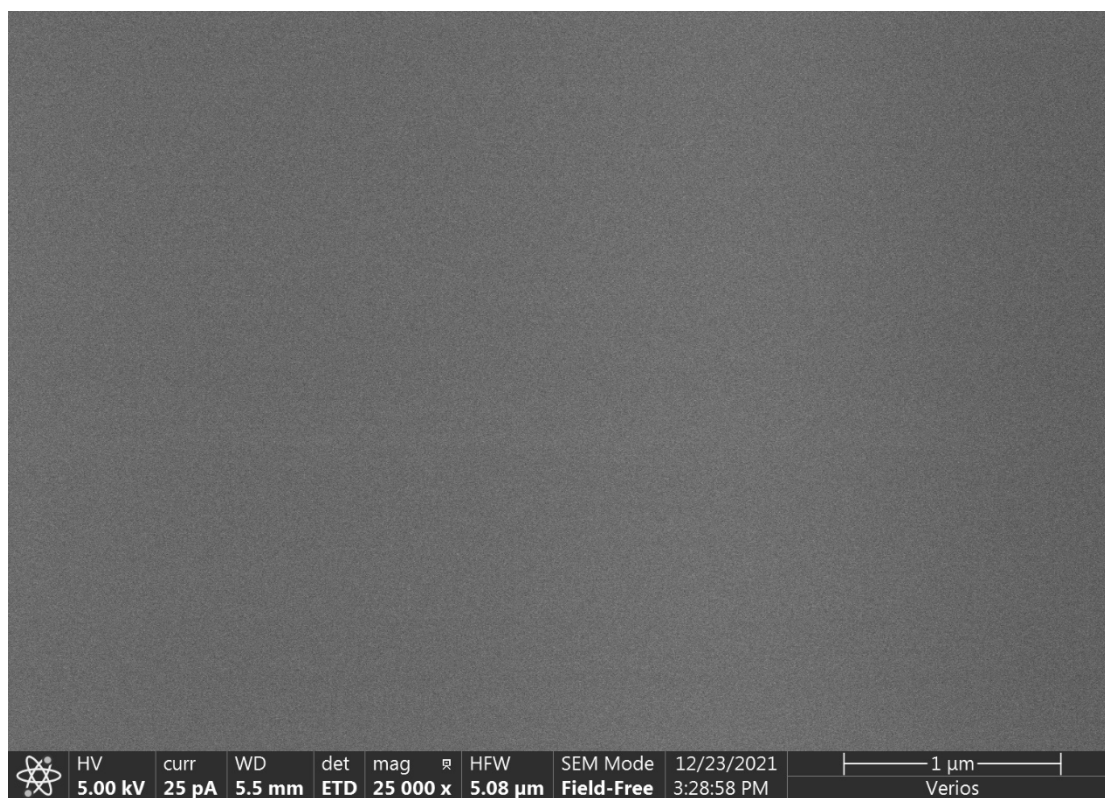

Figure S1: FESEM of silica aerogel/resin composites before adding silica aerogel filler.

Supplement: Supplementary file 1 [file molecules-27-04414-s001.zip › molecules-1807488-supplementary.pdf]
